# Otite moyenne aigue compliquée de pneumencéphalie et d'une méningite à pneumocoque

**DOI:** 10.11604/pamj.2015.22.389.8516

**Published:** 2015-12-30

**Authors:** Ali Derkaoui, Mohammed Khatouf

**Affiliations:** 1 Service d'Anesthésie-Réanimation, CHU Hassan-II de fes, Faculté de Médecine et de Pharmacie de Fès, Université sidi Mohammed ibn Abdellah, Fès, Maroc

**Keywords:** Otite média, Pneumencéphale, méningite, Otitis media, pneumencephalus, meningitis

## Image en medicine

La pneumencéphalie otogéne est une complication peu décrite dans la littérature. Elle est due à la genèse de micro fistule entre l'oreille moyenne ou postérieur et le crâne. Nous rapportons le cas d'une patiente de 65 ans qui s'est présentée dans notre formation pour trouble de conscience fébrile compliquant une otite moyenne. Le scanner cérébral a mis en évidence une pneumencéphalie au niveau de l'angle ponto-cérébelleux. L'analyse du liquide céphalorachidien a été en faveur d'une méningite purulente à pneumocoque. L’évolution a été favorable sous une antibiothérapie à base de céphalosporines de troisième génération associée à un traitement symptomatique.

**Figure 1:**
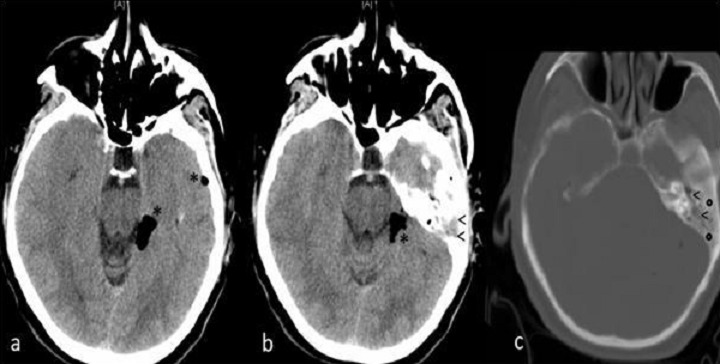
Coupes axiales à l’étage pétreux en fenêtrage parenchymateux (a et b) et osseux (c) montrant des bulles de pneumocéphalie péri-pétreuses gauches sans trait de fracture individualisable, cependant on note un comblement des cavités de l'oreille moyenne homolatérale associé à une ostéosclérose

